# MOF-Based Platform for Kidney Diseases: Advances, Challenges, and Prospects

**DOI:** 10.3390/pharmaceutics16060793

**Published:** 2024-06-11

**Authors:** Li-Er Deng, Manli Guo, Yijun Deng, Ying Pan, Xiaoxiong Wang, Govindhan Maduraiveeran, Jianqiang Liu, Chengyu Lu

**Affiliations:** 1 Department of Nephrology, Dongguan Traditional Chinese Medicine Hospital, Dongguan 523000, China; 2 Dongguan Key Laboratory of Drug Design and Formulation Technology, Guangdong Provincial Key Laboratory of Medical Molecular Diagnostics, Guangdong Medical University, Dongguan 523808, China; 3 School of Materials and Environmental Engineering, Shenzhen Polytechnic University, Shenzhen 518055, China; 4 Materials Electrochemistry Laboratory, Department of Chemistry, SRM Institute of Science and Technology, Kattankulathur 603 203, Tamil Nadu, India; maduraig@srmist.edu.in

**Keywords:** kidney diseases, MOF, diagnosis

## Abstract

Kidney diseases are important diseases that affect human health worldwide. According to the 2020 World Health Organization (WHO) report, kidney diseases have become the top 10 causes of death. Strengthening the prevention, primary diagnosis, and action of kidney-related diseases is of great significance in maintaining human health and improving the quality of life. It is increasingly challenging to address clinical needs with the present technologies for diagnosing and treating renal illness. Fortunately, metal-organic frameworks (MOFs) have shown great promise in the diagnosis and treatment of kidney diseases. This review summarizes the research progress of MOFs in the diagnosis and treatment of renal disease in recent years. Firstly, we introduce the basic structure and properties of MOFs. Secondly, we focus on the utilization of MOFs in the diagnosis and treatment of kidney diseases. In the diagnosis of kidney disease, MOFs are usually designed as biosensors to detect biomarkers related to kidney disease. In the treatment of kidney disease, MOFs can not only be used as an effective adsorbent for uremic toxins during hemodialysis but also as a precise treatment of intelligent drug delivery carriers. They can also be combined with nano-chelation technology to solve the problem of the imbalance of trace elements in kidney disease. Finally, we describe the current challenges and prospects of MOFs in the diagnosis and treatment of kidney diseases.

## 1. Introduction

The kidney is a vital organ in the human body that is essential for eliminating metabolites and preserving the stability of the internal environment. Owing to population aging and increasing the number of patients with diabetes and hypertension, the incidence of kidney diseases is rapidly increasing. Globally, the quantity of kidney disease patients is estimated to be ~600 million. The quantity of patients globally is growing at a rate of 6–7% per year [1]. An extremely high incidence of renal disorders places a heavy burden on global medical resources and poses a serious challenge to the prevention and control of kidney diseases worldwide. Therefore, kidney disease has become a major disease affecting the health of all human beings and a global public health problem [2].

Kidney diseases are diverse in type and cause. Acute kidney injury (AKI) is a serious disease in which metabolites accumulate in the body due to dysregulation of the glomerular filtration rate. It encompasses an unpredicted reduction in the glomerular filtration rate (GFR), creatinine evolution, decreased urine production, electrolyte turbulence, and uremia [3,4]. In real life, some kidney function recovery, a steady drop in glomerular filtration rate, and damage to the kidney tissue may lead to the start of chronic kidney disease (CKD). Diabetic kidney disease (DN) is a form of CKD [4]. Either CKD or AKI can further develop into end-stage renal disease (ESRD) or renal failure [5]. Overall, the etiology and types of kidney disease are extremely complex, but the final results are consistent with the disease status characterized by renal fibrosis and corresponding dysfunction [6]. Once the renal tissue is destroyed, it is a challenging task to restore renal function to the state of pre-disease [7]. When renal insufficiency occurs, the metabolic function of the kidneys is impaired. They cannot fulfill their blood purification function, and the uremic retention solutes are secreted by the kidneys [8]. High concentrations of uremic retention solutes are known as uremic toxins. Uremic toxins may be classified into three main courses based on their molecular weight and plasma protein binding characteristics [9,10]. The first is water-soluble small molecule compounds (Mw < 500 Da), including urea, creatinine, etc. The second is water-insoluble medium molecule compounds (Mw > 500 Da), such as β2-microglobulin, peptides, etc. The third category is protein-bound uremic toxins (PBUTs), which include hippuric acid (HA), indoxyl sulfate (IS), and p-cresol sulfate (pCS).

Currently, human therapeutic options for kidney diseases are nevertheless restricted. Dialysis and kidney transplantation are still the available therapeutic choices for most kidney disease patients [11,12]. Therefore, human beings need innovative approaches, drugs, and policies to diagnose and handle renal illnesses precisely, conveniently, and effectively. Nanotechnology, as a powerful driving force for biomedical progress, has revealed an excessive capacity for applications in the areas of disease bioengineering, early detection, therapy, and prevention [13,14,15]. The growing clinical applications of nanotechnology offer new solutions for the treatment of renal diseases [16,17]. The MOFs are a representative new version of porous and crystalline nanostructures, which are constructed with organic ligand-derived nodes of cluster or strong metal ion coordination [18]. The MOFs have a highly distinct surface area, tunable pore dimension and morphology, and unsaturated metal locations for coordination [19,20]. Consequently, MOFs are used in many different contexts, such as catalysis [21,22], chemical sensors [23], drug delivery [18], adsorbents [24], etc. Numerous research scientists have studied the utilization of MOFs in the diagnosis and treatment of kidney diseases. 

In the diagnosis of kidney diseases, MOFs are commonly used as sensors to detect biomarkers in human biological samples, and diseases are diagnosed based on the levels of these biomarkers. The porous nature of MOFs is advantageous for the enrichment of biomarkers by sensors, thereby improving the detection efficiency of sensors. In terms of treatment for kidney diseases, MOFs not only serve as adsorbents for uremic toxins in blood dialysis but also act as delivery carriers for therapeutic drugs. They can load a large amount of drugs and target them to the kidneys. Additionally, MOFs can also be used to supplement essential trace elements in the body to address certain kidney diseases caused by deficiencies in trace elements. In conclusion, MOFs demonstrate great potential in both the diagnosis and treatment of kidney diseases.

Therefore, the present review summarizes the research progress of MOFs in the diagnosis and treatment of renal diseases. We also discussed the existing problems, challenges, and future development directions for the diagnosis and action of kidney disease. The present review aims to provide a reference for further research and clinical application of MOFs in the field of kidney disease. Through this review, we hope to emphasize the potential application value of MOFs as an innovative material in the field of kidney disease and provide theoretical support and enlightenment for their future research and clinical transformation.

## 2. Application of MOFs in the Kidney Diseases Diagnosis

**Figure 1:**
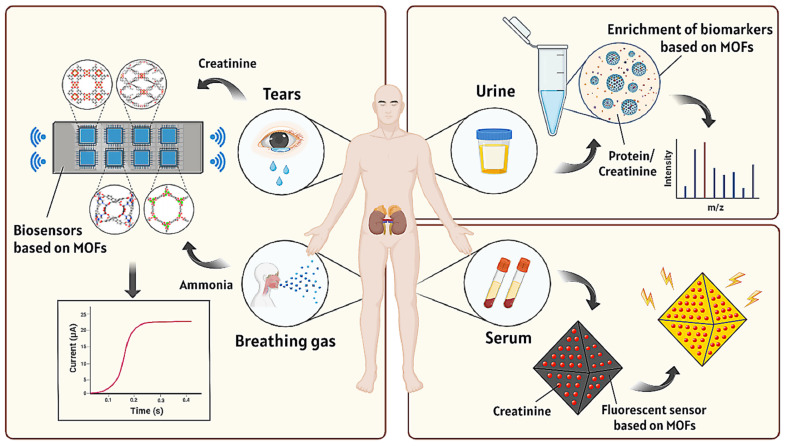
Diagnosis mechanism diagram of kidney disease based on MOFs.

The main approach to diagnosing kidney diseases based on MOFs is to utilize the porous adsorption properties of MOFs to enrich biomarkers associated with kidney diseases. Biomarkers are usually abnormal in the early stages of the disease, which helps with early detection and improves the success rate of treatment. Different biomarkers can correspond to different types of diseases or different subtypes of the same disease, thus helping doctors develop more precise treatment plans. In addition, biomarkers can also predict disease progression, dynamically monitor changes in disease, and track treatment effects in real-time. In conclusion, the detection of biomarkers is crucial for disease management. MOFs are usually designed as biosensors for detecting various biomarkers related to kidney diseases in human biological samples (Figure 1). 

### 2.1. Biomarkers in Urine

Urine plays an important role in the physiological and pathological circumstances of the kidney. Urinalysis is a very attractive in vitro test with the advantages of being completely noninvasive, easy to sample, and low cost, making it a perfect body fluid for nursing kidney disease [25,26]. Urine is a waste product that can vary over time without significantly breaking down proteins. It is a valuable tool for the sensitive and early identification of kidney disease indicators [27]. Recently, urinary metabolite outlining was used to disclose urinary metabolic evidence for sensing diseases [28,29].

**Figure 2:**
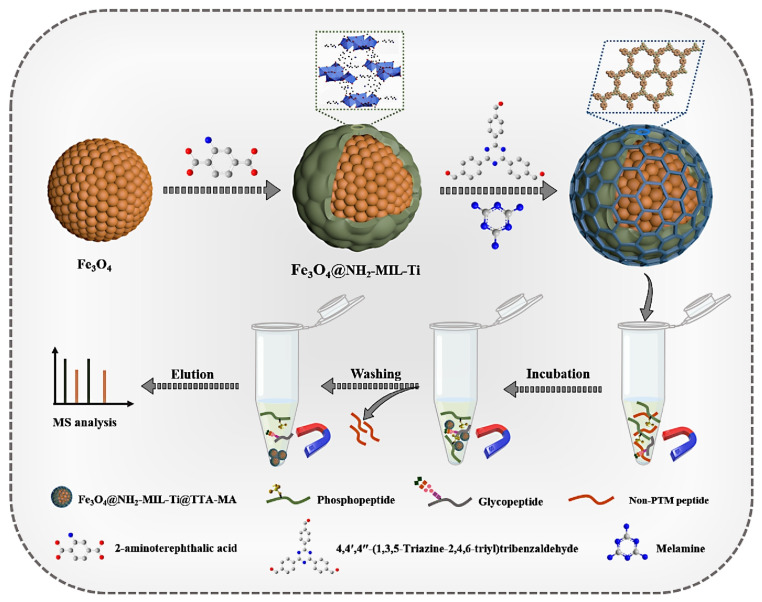
Pictorial representation of the preparation strategy for Fe_3_O_4_@NH_2_-MIL-Ti@TTA-MA and its improvement flow chart of glycopeptide/phosphopeptide. Reproduced from ref. [44] with permission from Elsevier, copyright 2023.

Creatinine level is a reliable biomarker in urine for assessing renal function [30]. Surface Enhanced Raman Scattering (SERS) plays a crucial role in the field of biochemical analysis due to its unique ability to identify molecular fingerprints and is a promising technique for measuring urinary creatinine levels [31,32]. To improve the sensitivity of SERS detection, researchers explored the preparation of composite SERS substrates with strong affinity for target molecules. A MOF-SERS platform has been reported for trace detection, which can selectively capture target molecules [33,34]. Jiang et al. prepared Au@MIL-101(Fe) composites through the in-situ growing of Au nanomaterials using MIL-101 (Fe) for monitoring creatinine (Figure 2) [35]. Using electrostatic forces, creatinine molecules can be enriched into the porous structure of MIL-101(Fe) and in close proximity to gold nanoparticles, which significantly improves the Raman scattering signal. The limit of detection (LOD) of the composite for creatinine in human urine is measured to be ~0.1 μmol·L^−1^. Therefore, Au@MIL-101(Fe) demonstrates the combined advantages of high sensitivity, selectivity, stability, and strong immunity to interference in the field of biomarker detection.

In addition, protein in urine is also a biomarker closely related to kidney diseases [36,37]. Post-translational modifications (PTMs) of proteins, including phosphorylation, glycosylation, acetylation, and ubiquitination, have garnered a lot of attention in recent years [38,39,40]. However, the low concentration and extremely complex composition of glycoproteins in urine samples make them problematic to analyze directly. It is worth drawing attention to the fact that a series of hydrophilic hybrid materials based on magnetic MOF groups have been well-developed in recent years. For example, Lu and colleagues developed a maltose-modified magnetic MOF for efficient enrichment of N-chain glycopeptides [41]. Liu et al. prepared mMOF@Au@GSH by attaching glutathione on Au-immobilized MOF to identify glycopeptides in human serum [42]. However, the intrinsic hydrophilicity of MOFs with magnetic properties alone occasionally fails to encounter the necessities of protein examination, particularly when dealing with complex examples.

Therefore, Hu et al. prepared glucose-6-phosphate (G6P)-based magnetic UiO-66-NH_2_ complexes (labeled Mag Zr-MOF@G6P) using a simple one-step modification strategy [43]. Through hydrophilic contact separation techniques, Mag Zr-MOF@G6P, a hydrophilic carbohydrate, can be used to capture and differentiate glycopeptides. The Mag ZrMOF@G6P composite offers an ultra-low detection limit (down to 0.1 fmol/μL), good selectivity (mass ratio of HRP enzymatic product to BSA enzymatic product up to 1:200), high binding ability, and countless capacity for reuse for glycopeptide improvement. This is due to the improved hydrophilicity, suitable porous structure, great precise surface area, high stability, and fast magnetic retort of the Fe_3_O_4_ core. This study revealed that 13 primitive glycoproteins derived from urinary glycopeptides of patients were significantly involved in a variety of cancer-related events, such as the binding of collagen, immunoglobulin receptors, antigens, and complement activation procedures. This Mag Zr-MOF@G6P composite has been successfully applied to the comprehensive proteomic analysis of glycopeptide sequences, sites of glycosylation, and original glycoproteins in the urine of fit humans and patients with renal cancer.

In addition, Xiong et al. fabricated MOF@COF (COF: covalent organic framework) hybrid material, Fe_3_O_4_@NH_2_-MIL-Ti@TTA-MA, which achieves a ‘super-merger’ by covalently integrating two encouraging porous crystalline materials onto a magnetic core [44]. The Ti-O group in MOF (NH_2_-MIL-Ti) and the great hydrophilicity of COF (TTA-MA) give the synthesized permeable hybrids excellent hydrophilicity. Therefore, Fe_3_O_4_@NH_2_-MIL-Ti@TTA-MA may be used as a hydrophilic interaction chromatography (HILIC) adsorbent for the enrichment of glycoproteins. The metal oxide affinity chromatography (MOAC) adsorbent is used to enrich phosphoproteins by utilizing the adsorbing characteristics of Ti-O with phosphate groups. More significantly, Fe_3_O_4_@NH_2_-MIL-Ti@TTA-MA allows the efficient concurrent improvement of glyco- and phospho-proteins. Four phases make up the enrichment protocol, including loading, washing, elution, and mass spectrometry (MS) analysis (Figure 2). This porous hybridized material retains the structural characteristics of both MOF and COF while exhibiting enhanced performance under the synergistic effect of the two. This is superior to the effect of each when used alone. The excellent ability of the porous hybridized material to enrich both glyco- and phospho-proteins which provides the possibility for further enhancement of glyco- and phospho-proteins in practical complex biological samples. The experimental data show that Fe_3_O_4_@NH_2_-MIL-Ti@TTA-MA has a detection limit as low as 0.2 fmol for glycoproteins and 0.04 fmol for phosphoproteins, both of which exhibit very high sensitivity. It is also highly selective, distinguishing HRP or β-casein from BSA (bovine serum albumin) at a ratio of 1:1000. In addition, Fe_3_O_4_@NH_2_-MIL-Ti@TTA-MA can be reused at least five times for good durability. In conclusion, the synthesized magnetic MOF@COF can distinguish patients with renal disease syndrome according to the number of enriched glyco- and phospho-proteins and has an extremely low detection limit, good selectivity, and good reusability.

**Figure 3:**
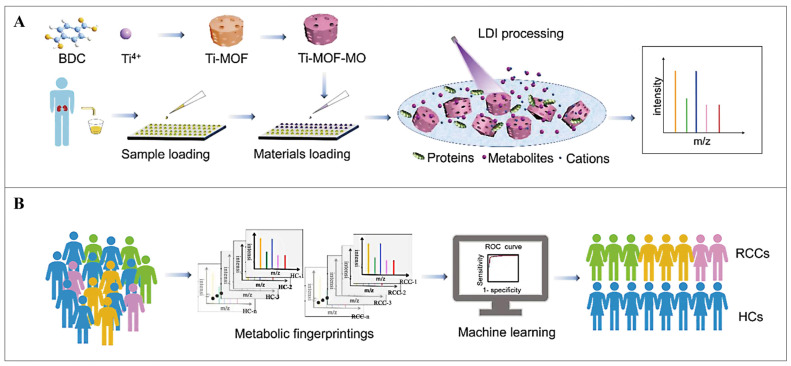
(**A**) Scheme for creation and application of Ti-MOF-MO-aided LDI-MS, workflow diagram of examining urine metabolites. (**B**) RCCs sensor using metabolic fingerprinting and ML. Reproduced from ref. [49] with permission from American Chemical Society, copyright 2022.

Renal cancer is a common type of malignant tumor in adults, with a relatively high proportion of renal cell carcinoma (RCC) [45]. Metabolic alterations are strongly related to a diversity of diseases, including RCC [46,47]. Urine is considered to be the most capable liquid to deliver molecular variants for RCC detection [48]. Hu et al. constructed metal oxides (Ti-MOF-MO) with porous structures derived from MOFs to contribute to the laser desorption ionization mass spectrometric (LDI-MS) method with high sensitivity, high throughput, and rapidity for urinary metabolite analysis (Figure 3) [49]. This Ti-MOF-MO cleverly inherited the surface structure and sponginess of MOFs, combined with the laser adsorption ability of metal oxides and solved the cumbersome pre-processing problem required by LDI-MS for analyzing urinary metabolites (such as Arg, His, Glu, etc.). This technique effectively separated papillary RCC (pRCC), chromophobe RCC (chRCC), and clear cell RCC from healthy controls (HCs). It had a strong recognition ability in stages I and II of RCC, which contributed to the reduction of mortality. Overall, this is an analytical method with the characteristics of fast analysis, minimal sample consumption (only 5 μL), easy operation, and non-invasiveness, which not only successfully distinguished kidney cancer but also achieved renal cancer typing, staging, and tumor size (threshold 3 cm) identification. It is expected to provide an effective large-scale detection tool for cancers, including RCCs.

In summary, by optimizing and innovating the design of MOF materials, researchers have greatly improved the accuracy and depth of urinalysis and provided a powerful tool for the early diagnosis and monitoring of kidney diseases and other related diseases.

### 2.2. Biomarkers of Respiratory Gases

An efficient and least invasive technique for diagnosing disease is emerging by analyzing volatile organic compounds (VOCs) and gaseous inorganic molecules formed endogenously in the human respiratory system [50]. Respiratory samples possess the advantages of non-invasive, continuous availability, allowing simple and rapid sampling and real-time monitoring [51]. Disease detection and monitoring through respiratory gases has unique and untapped clinical potential. Ammonia is a recognized biomarker in the known concentration range of healthy people and CKD patients [52,53]. Elevated quantities of nitrogenous metabolic waste are produced in the body by the patients of CKD imbalanced equilibrium concentrations of urea and ammonia. Salivary urease breaks down urea into ammonia, and salivary ammonia vaporizes into a gaseous medium that is expelled through breathing [54,55,56]. It showed that respiratory ammonia altitudes are associated with levels of blood urea nitrogen [57]. There is a high relationship between respiratory ammonia levels and levels of blood urea nitrogen in hemodialysis patients before and after dialysis [56,58,59]. The determination of exhaled ammonia levels represents a potential noninvasive method for evaluating body ammonia levels [60]. Therefore, the detection and monitoring of respiratory gases hold promise as a research approach for diagnosing kidney diseases.

**Figure 4:**
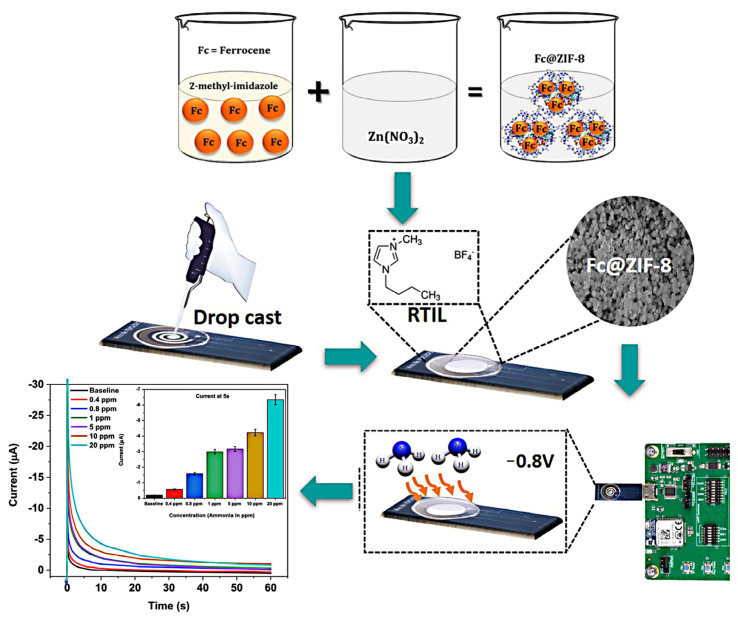
ZIF-based electrochemical nose (ZENose) platform. Reproduced from ref. [62] with permission from American Chemical Society, copyright 2021.

Typically, biomarker gases are present in exhaled gas only in trace amounts, and their adsorption in MOFs follows Henry’s law. Day et al. designed a MOFs-based gas sensor array (electronic nose) based on an improved Henry’s coefficient (CLAC) calculation method for the detection of ammonia biomarkers in kidney diseases [61]. This is a method with low computational requirements while maintaining sufficient accuracy. The researchers used this device to successfully quantify ammonia levels in fit and unfit breath testers, demonstrating the potential of such devices in the detection of kidney disease. In addition, Banga et al. established a gas sensor platform using an electrochemical strategy (electrochemical nose system, ZENose) for real-time detection of ammonia levels in respiratory gases to evaluate renal function. The system encapsulates the Faraday probe (ferrocene, Fc, as redox medium) into ZIF-8 with excellent physical adsorption characteristics (Figure 4) [62]. The real-time electrochemical sensor possessed high sensitivity and specificity for trace ammonia (up to 400 ppb). The anode current of the sensor increased proportionally as the ammonia concentration increased from 400 ppb to 20 ppm, indicating good responsiveness of the sensor. For a respiratory omics platform, this is an electrochemical microelectronic platform for the first time for quick, dynamic, and non-invasive sensing of gaseous ammonia.

Both Day’s team and Banga’s team’s research focus on the use of MOFs to develop novel sensors that can noninvasively detect ammonia, a biomarker in exhaled gas [61,62]. These studies not only advance the early noninvasive diagnosis of kidney diseases but also provide new ways to reduce mortality. Although the gas detection technology based on MOFs still needs to be further improved, such as expanding the types of gases to be detected, correcting for the effect of humidity, and optimizing the effect of force fields, it has significantly improved the efficiency of detection and provided an innovative direction for future non-invasive disease monitoring.

### 2.3. Biomarkers in Other Samples

**Figure 5:**
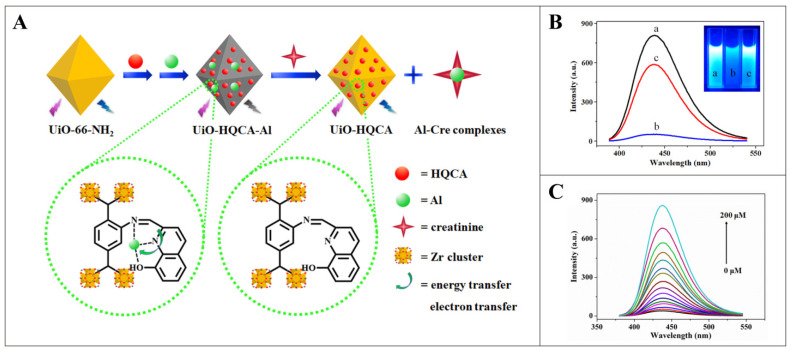
(**A**) Fluorescence induction mechanism diagram of UiO-HQCA-Al for creatinine. (**B**) Fluorescent emission spectra of (a) UiO-HQCA and UiO-HQCA-Al in the (b) absence and (c) presence of 150 μM Cre (incubation time of 1 min). Inset: photographs taken under 365 nm UV lamp. (**C**) Fluorescence spectrum of UiO-HQCA-Al after adding different concentrations of creatinine. Reproduced from ref. [67] with permission from Elsevier, copyright 2020.

The serum creatinine level is an important index to measure the health status of the human kidney. Fluorescence sensing technology is a powerful technology for the detection of creatinine. Owing to its good fluorescence characteristics and abundance of unsaturated coordination metal active sites (Zr), UiO-66 is regarded as one of the most attractive possibilities for fluorescent materials [63,64]. As a fluorescent probe, UiO-66 needs to have its specificity and sensitivity improved. Post-synthesis modification (PSM) allows the introduction of suitable recognition sites without altering the MOF topology [65,66]. Qu et al. successfully developed a fluorescence-enhanced MOF sensor for the detection of creatinine [67]. This sensor was synthesized by post-synthetic modification (PSM) of UiO-66-NH_2_ using 8-Hydroxy-2-quinolinecarboxaldehyde (HQCA) and Al^3+^, which achieved a strong Lewis acid-base interaction between Al^3+^ and creatinine. In addition, the developed sensing system is based on a turn-on sensing mechanism to detect creatinine. Specifically, the UiO-HQCA fluorescence quenched by Al^3+^ can be restored by creatinine (Figure 5A). The sensor has high sensitivity (detection limit of 4.7 nM), a wide linear range (0.05~200 μM), a fast response time (1 min), and high selectivity for creatinine (Figure 5B,C). Its utility has been demonstrated by measuring creatinine in human serum samples, and it has broad application prospects.

**Figure 6:**
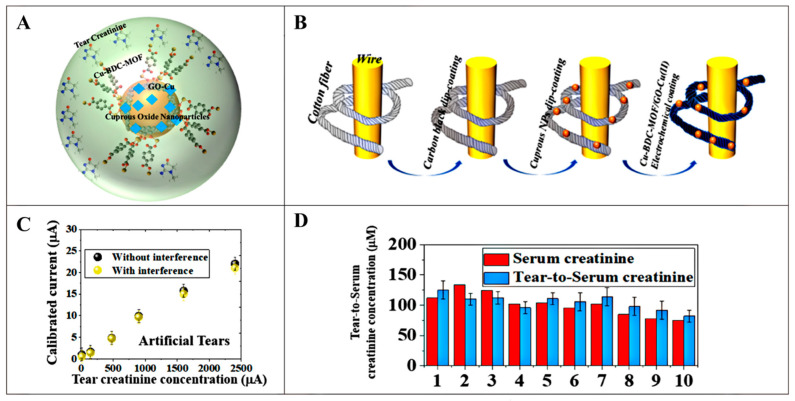
(**A**) Schematic representation of Cu-BDC MOF/GO-Cu(II) and embedded Cu_2_O nanoparticles, and (**B**) its electrode preparation for sensing creatinine in tear samples. (**C**) Calibration plot of measured current response against the creatinine concentration. (**D**) Assessment of sensing serum creatinine in hospital and tear-to-serum creatinine. Reproduced from ref. [71] with permission from American Chemical Society, copyright 2021.

However, the determination of serum creatinine level not only requires a skilled technician to obtain a blood sample but also takes a long time to obtain the test result [68,69]. Fortunately, it has been reported that creatinine content in tears is positively correlated with blood creatinine levels, and thus, tears can be a good biomarker for assessing kidney function [70]. In recent years, wearable sensors have been greatly developed, and a fiber-based eyeglass sensing device for selective detection of tear creatinine has been invented [71]. The device combines copper-containing carboxylic acid (BDC) MOF with graphene oxide (GO)-Cu (II) and integrates it on cuprous oxide nanoparticles (Cu_2_O NPs), and the ternary complex of Cu-BDC MOF/GO-Cu(II)/Cu_2_O NPs was used for creatinine detection (Figure 6A,B). This design skillfully facilitated the diffusion of creatinine molecules from tear fluid into its porous structure and their permanent capture for noninvasive detection of serum creatinine. The composite sensor demonstrated excellent specificity, maintaining recognition efficiencies of up to 95.1% over the creatinine concentration range of 1.6 µM to 2400 µM, even in the presence of interfering agents such as dopamine, urea, and uric acid (Figure 6C). Through machine learning algorithms, the device was able to effectively differentiate between normal and abnormal (low, medium, and high) serum creatinine status based on tear creatinine levels with an accuracy of 83.3%. In practice, the eyewear sensor showed a low mean deviation between tear creatinine and serum creatinine values measured in clinical laboratories (Figure 6D), demonstrating its effectiveness in differentiating between patients with normal renal function and those with chronic kidney disease (creatinine concentration > 1000 µM). This miniaturized and portable ophthalmic detector greatly enhances the convenience of testing creatinine and follows the trend of telemedicine services, making personal health monitoring more autonomous and convenient.

The detection of biomarkers is a very effective way to monitor kidney health. The advent of MOFs has greatly advanced this approach. Researchers have developed a variety of MOF-based biosensors to detect biomarkers in biological samples, thereby improving the ability to diagnose kidney diseases early. Table 1 shows the advantages and limitations of various MOF-based agents for the diagnosis of kidney diseases. Although these agents are now only at the laboratory stage, there has not been large-scale clinical validation, but their potential application prospects are very good. It is believed that in the future, these agents will play an important role in the clinical diagnosis of kidney diseases.

## 3. Application of MOFs in Kidney Disease Treatment

In the field of treatment for kidney diseases, MOFs and their derivatives have shown unprecedented potential due to their structural characteristics, selectivity, and biocompatibility advantages. This chapter will explore in depth the different types of MOFs, including Zr-based, Fe-based, and other types of MOFs, and how they act as adsorbents for uremic toxins in the treatment of kidney diseases. It will also explore their potential applications in drug delivery and discuss nanoscale chelation technology solutions for addressing trace element imbalance issues.

### 3.1. MOFs as a Uremic Toxin Adsorbent for the Kidney Disease Treatment

The growth of various uremic toxins in blood samples caused by renal metabolic dysfunction is likely to lead to fatal renal failure in patients. Because of their insignificant dimensions and water solubility, urea and creatinine are the hardest poisons to eliminate. In addition, PBUTs bind to human serum albumin (HSA) via a variety of relationships, including hydrophobic communication, electrostatic interface, and van der Waals force [72,73,74]. Such strong interactions result in the difficult removal of PBUTs from the blood. Therefore, the effective elimination of excessive uremic toxins in the blood is essential. Presently, the most frequently employed approach to remove uremic toxins from the body is hemodialysis. Diffusion, convection, and adsorption are the fundamental concepts of hemodialysis and the separation of uremic toxins from proteins. The blood cells are achieved by means of a semipermeable membrane and the upkeep of electrolyte and acid-base equilibrium in the human body [75].

**Figure 7:**
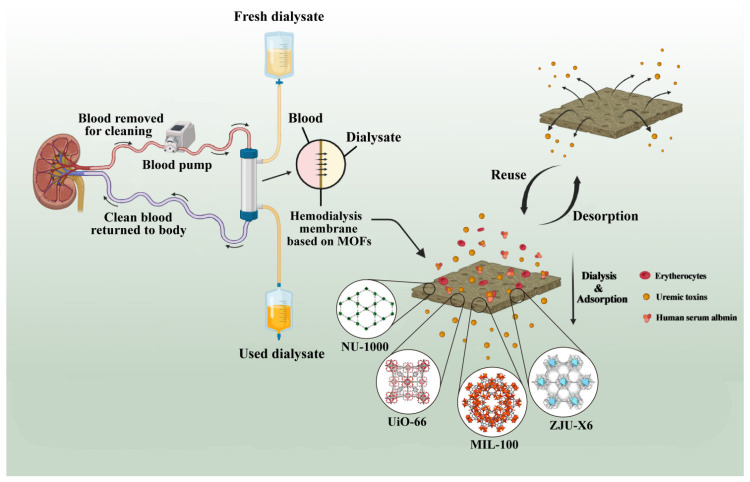
Application mechanism of MOFs as uremic toxin adsorbent in the treatment of kidney disease.

With the development of technology, the selectivity of dialysis membranes has improved, but convection and diffusion methods still have the drawbacks of inconvenience and cost [76]. Consequently, researchers have focused more on eliminating uremic toxins from patients’ blood by improving the adsorption ability of dialysis membranes. In a typical hemodialysis device, uremic toxins need to be removed through a dialysate regeneration process to use fresh dialysate [77]. Also, desorption of uremic toxins from dialysis membranes and regenerative reuse of dialysis membranes are necessary. The system of dialysate regeneration uses nanoporous adsorbents, which are typically necessary to achieve mobility in hemodialysis [78,79]. Novel porous nanomaterials of MOFs and their derivatives and complexes show great potential in uremic toxin adsorption. This means that the limitations of zeolites, composite membranes, activated carbon, and other traditional dialysis materials for adsorption have been overcome. The utilization of MOFs as uremic toxin adsorbents for the treatment of kidney diseases is shown in Figure 7.

#### 3.1.1. Zr-Based MOFs

Zr-based MOFs have relatively superior chemical and thermal stability, which can be used in different application fields. NU-1000 (NU: Northwestern University) is a representative of Zr-based MOFs composed of Zr_6_ clusters and 1,3,6,8-tetrakis (*p*-benzoic-acid)pyrene (H_4_TBAPy) [80]. The pyrene-based MOF NU-1000 had the highest toxin elimination effectiveness among the numerous Zr-based MOFs [81]. At 303 K, the maximum adsorption capacities of NU-1000 for pCS and IS are 440 mg·g^−1^ and 193 mg·g^−1^, respectively. Furthermore, the removal efficiency of NU-1000 for pCS can reach up to 94%. The superior performance of NU-1000 in uremic toxin adsorption can be attributed to the porous morphology and customizable dimension screening of MOFs [82,83,84]. However, the tightly packed structure and poor hemocompatibility of the small-sized NU-1000 affect its rapid clearance of PBUTs, limiting its clinical application [85,86,87]. Chao et al. utilized the nanoporous structure of pollen (Pol) in conjunction with polydopamine mediation (PP) to fabricate a NU-1000 nanoparticle (PPNU) possessing a robust adsorption capacity for PBUTs [88]. Subsequently, the PPNU was functionalized with heparin (PPNUH), leading to a significant enhancement in the blood compatibility of NU-1000. Meanwhile, PPNUH maintained the high adsorption capacity of NU-1000 for PBUTs (282 mg·g^−1^ for pCS, 329 mg·g^−1^ for IS, and 188 mg·g^−1^ for HA). Moreover, in duplicate blood perfusion, PPNUH can quickly adsorb 85% of free PBUTs in 10 s and remove 70% of albumin-bound PBUTs within 1 min. PPNUH’s delicate structure demonstrates safe, fast, and efficient PBUT removal.

UiO-66 (UiO: University of Oslo) is another illustration of Zr-derived MOF materials prepared with Zr_6_O_4_(OH)_4_ clusters and terephthalate linkers [89]. Among a series of MOF materials, UiO-66 is promising because of its high thermal stability and chemical stability [90]. On the original UiO-66, the maximum removal efficiency of 1.5 mg UiO-66 adsorbed in 0.1 mM p-cresol potassium sulfate, potassium sulfate, and hippuric acid was 2.1%, 21%, and 90%, respectively [81]. However, the morphology of UiO-66 materials has almost no defects, which may significantly reduce the adsorption capacity [91]. Based on this, Dymek et al. focused on optimizing the preparation of imperfect UiO-66 to attain an effectual uremic toxin adsorbent [90]. Through the functionalization of 1,4-benzenedicarboxylic acid connectors with NH_2_ moieties to increase structural defects and electronic properties. They acquired a series of UiO-66 with adjusted structures. The prepared UiO-66-NH_2_ (75%) and UiO-66-NH_3_ (75%) 12.5% HCl have the largest adsorption capacity for 3-indoleacetic acid and hippuric acid. Moreover, both UiO-66-NH_3_ (75%) and UiO-66-NH_2_ (75%) 12.5% HCl had good renewable utilization properties, with UiO-66-NH_3_ (75%) retaining about 80% of 3-indoleacetic acid removal efficiency after three adsorption cycles; and UiO-66-NH_2_ (75%) 12.5% HCl, with the second and third cycles. The maximum adsorption was ~76% and ~70%, respectively. Therefore, UiO-66 is a potential candidate for adsorption of uremic toxins [90].

**Figure 8:**
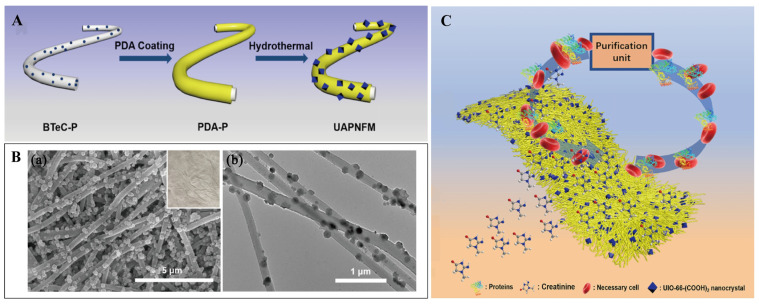
(**A**) Scheme for the fabrication of UAPNFM. (**B**) SEM (a) and TEM (b) images of UAPNFM. (**C**) The dialysis procedure scheme is based on UAPNFM. Reproduced from ref. [97] with permission from Elsevier, copyright 2022.

UiO-66-(COOH)_2_ contains a huge quantity of oxygen-containing organic moieties such as -COOH and -OH moieties. The structure is stable and is expected to adsorb creatinine [92,93]. However, because of their crystal nature, MOFs appear in powder form, thus preventing their practical application in the adsorption process [94]. To improve the practicability of MOFs, scientists combined MOFs with a polymer matrix as an adsorbent, showing good adsorption and reusability [95,96]. Abdelhameed and his colleagues directly grew UiO-66-(COOH)_2_ in cotton fabric. The synthesized UiO-66-(COOH)_2_@cotton composite showed high creatinine adsorption capacity and recyclability [95]. The highest probable adsorption levels of the original fabric, non-in-situ composites, and in-situ composites were 113.6, 192.3, and 212.8 mg·g^−1^, respectively. The study suggested that the regeneration of the creatinine adsorption composite was effectively achieved using the methanol ultrasonic cleaning technique. During the evaluation of the regeneration performance, it was discovered that the initial maximum adsorption ability of the material for creatinine was ~261.3 mg·g^−1^. After undergoing the first regeneration cycle, the capacity of adsorption was decreased to 242.7 mg·g^−1^, which maintained about 91% of the initial adsorption efficacy. In the third regeneration cycle, the adsorption capacity was decreased to 218.7 mg·g^−1^, which still maintained about 82% of the original adsorption efficiency. This indicates that even after three repeated regenerations, the ability of the composite to remove creatinine decreased only slightly, by only about 16%. Also, Li et al. attached UiO-66-(COOH)_2_/PAN to build a nanofiber membrane (Figure 8A) [97]. The anchoring content was 54.99 wt%. The UAPNFM was positioned in the space between two dialysis chambers to create crossflow during the procedure by mimicking the opposing blood and dialysate flow directions. Owing to the composite nanofiber membrane’s porous shape and the osmotic pressure produced by the crossflow, some creatinine molecules were adsorbed on the UAPNFM while others migrated from the simulated blood into the dialysate flow (Figure 8C). The UAPNFM facilitated the adsorption of creatinine by bonding hydrogen, electrostatic approaches, and interactions of π-π. The maximal creatinine adsorption capacity of UAPNFM was 168.63 mg·g^−1^. The creatinine clearance was 226 mL·min^−1^ at a simulated blood flow rate of 200 mL·min^−1^, 1.24 times greater than that of the commercially available FX60 dialyzer (182 mL·min^−1^). Following four hours of simulated dialysis, 82.48% of the UAPNFM for creatinine was cleared, while 93.09% of the protein in the bovine serum albumin (BSA) was retained. Moreover, following four cycles, the creatinine elimination efficiency held steady at 82.31%. In conclusion, these UAPNFM nanocomposites exhibited good blood compatibility, effective uremic toxin removal, excellent protein retention, and good regeneration ability.

**Figure 9:**
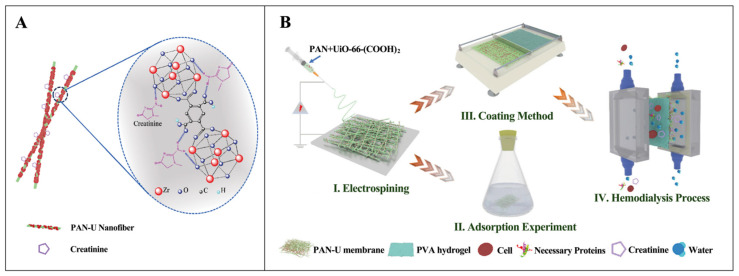
(**A**) Adsorption mechanism, and (**B**) Scheme for the construction process of PAN-U nanofibrous membrane for the adsorption of creatinine. Reproduced from ref. [98] with permission from Elsevier, copyright 2021.

Furthermore, Ding et al. prepared reticulated nanofibrous affinity membranes for the adsorption of creatinine by stringing synthesized UiO-66-(COOH)_2_ nanostructures onto polyacrylonitrile nanofibers using the colloidal electrostatic spinning procedure [98]. The prepared PAN/UiO-66-(COOH)_2_ nanofiber membranes had an optimal UiO-66-(COOH)_2_ loading (60 wt%). PAN-U-60 had a worthy ability for creatinine adsorption, with a maximum capacity of adsorption up to 54 mg·g^−1^. The creatinine adsorption on PAN-U-60 nanofiber membranes could be achieved by UiO-66-(COOH)_2_ oxygen-present functional moieties, such as -OH and -COOH, and hydrogen bonds formed by the interaction between amino and carbonyl functional groups contained in creatinine (Figure 9A). On this basis, the researchers prepared new dialysis and adsorption processes for nanofiber composite membranes comprising a PAN-U-60 nanofiber adsorbent layer and a polyvinyl alcohol (PVA) hydrogel separator layer by using an encapsulation reaction method (Figure 9B). Precisely, the top cover was a PVA hydrogel thin coating, which enabled rapid dialysis of toxins through the cortex and prevented leakage of melanin. In the meantime, the bottom layer consisted of a PAN nanofiber matrix encapsulated with UiO-66-(COOH)_2_ nanostructures, which represented an adsorbent and sustenance for the dialyzed toxin, allowing it to be trapped in the dialysate. Toxins from blood and dialysate can be removed and adsorbed using this PVA/PAN-U TFNC composite membrane, which combines the benefits of both adsorption and dialysis. During simulated dialysis, the PVA/PAN-U-60 TFNC membrane provides substantial toxins removal from the blood (creatinine removal of 62.8%) while maintaining ultra-high protein retention (up to 98%). In addition, the volume of dialysate used for dialysis with PVA/PAN-U-60 TFNC membranes is merely a tenth that of PVA/PAN TFNC membranes at analogous dialysis enactment, significantly reducing the volume of dialysate. This is fully attributed to the creatinine adsorption by the PAN-U-60 nanofiber membrane, permitting the dialysate to be sanitized.

Zr-based MOFs are proven to demonstrate virtuous request prospects in the adsorption of uremic toxins. However, metal zirconium may be biologically toxic, which limits its commercial application [99].

#### 3.1.2. Fe-Based MOFs

Metal iron is a necessary ingredient for human health and possesses very little biological harm [100]. Water-stable MOFs that are offered commercially. Fe-based MOFs have good biocompatibility and are of countless importance for clinical applications. MIL-100 (Fe) (MIL = Materials of Institut Lavoisier) is a crystalline three-dimensional trimeric acid Fe^3+^ consisting of metal junction Fe^3+^ and organic linker 1,3,5-triphenyl tricarboxylic acid [101]. MIL-100 (Fe) as an adsorbent has the advantages of non-toxicity [102], stability under physiological conditions [103], and unique nanochannels that only molecules with low molecular weight can enter [104]. Moreover, the bulky specific surface area of MIL-100 (Fe) creates MIL-100 (Fe) have great prospects for creatinine adsorption [101,105].

**Figure 10:**
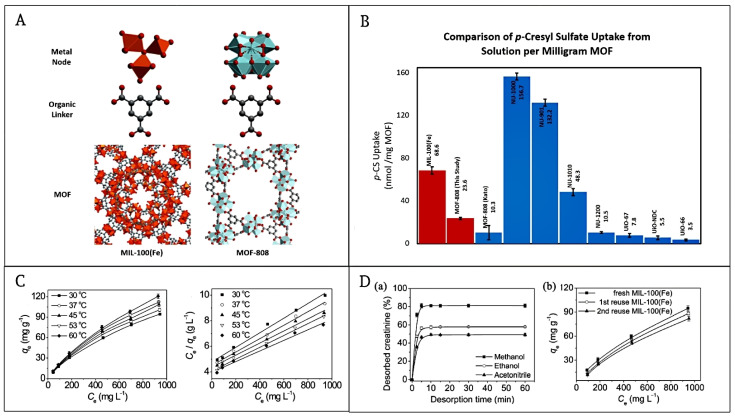
(**A**) The framework, organic linker, and metal node chemical structures of MIL-100(Fe) and MOF-808. (**B**) Relationship of uptakes with various MOFs. Reproduced from ref. [106] with permission from American Chemical Society, copyright 2020. (**C**) Adsorption of creatinine. (**D**) Desorption and regeneration of MIL-100 (Fe) for reuse. Reproduced from ref. [108] with permission from Royal Society of Chemistry, copyright 2014.

Cuchiaro et al. produced MOF-808 (Zr) and MIL-100 (Fe) with identical BTC molecules but distinct metal centers in the Fe node and the Zr node, respectively (Figure 10A) [106]. In MOF-808, Zr(IV) centers produced Zr_6_O_8_ clusters linked by -OH and BTC anions, while the centers of Fe^2+/3+^ exhibited an octahedral shape and formed oxocentered trimers covered by BTC anions. It is possible that distinct metal centers in comparable MOFs led to varying adsorptive tendencies toward PBUTs, even though their connectivity was similar. The quantity of adsorption that MIL-100 (Fe) showed in the absorption of pCS was 68.6 nmol·mg^−1^, significantly greater than that of MOF-808 (23.6 nmol·mg^−1^) (Figure 10B). It was hypothesized that Fe^2+/3+^ variable valence d-orbitals would take electrons from pCS’s sulfate more readily than Zr(IV), leading to a stronger combination. They speculated that the greater adsorption capacity of MIL-100 (Fe) than that of MOF-808 might have originated from the more favorable straight synchronization of pCS to empty sites of metal in MIL-100 (Fe).

Furthermore, regarding the absorption of urea and creatinine at 1 bar and 310 K, MIL-100 (Fe) performed better than traditional dialysis adsorbents, including activated carbon, zeolites, and polymeric materials [107]. Horcajada et al. synthesized two types of MILs, MIL-100 (Fe) and MIL-53 (Fe), and examined their adsorption of urea and found that these urea uptakes in MOFs, which could be higher than 692 mg·g^−1^ and 635 mg·g^−1^, respectively [103]. Also, MIL-100 (Fe) is a very promising adsorbent for the removal of creatinine and the adsorption isotherm of creatinine on MIL-100 (Fe) (Figure 10C) [108]. The creatinine adsorption on MIL-100 (Fe) increased gradually with the upsurge of primary creatinine concentration, indicating that MIL-100 (Fe) possessed respectable adsorption properties for high concentrations of creatinine. The adsorption capacity of MIL-100 (Fe) for creatinine was up to 190.5 mg·g^−1^ at physiological temperature (37 °C). The adsorption of creatinine on MIL-100 (Fe) was extemporaneous and heat-absorbing, which was mainly achieved by weak ligand interactions. The MIL-100 (Fe) adsorption capacity was decreased in the presence of HSA, which may be due to the presence of HSA on the MIL-100 (Fe) surface due to competitive adsorption [108]. When HSA (40 mg·mL^−1^) was present, the equilibrium adsorption capacity of creatinine on MIL-100 (Fe) decreased to 13.6 mg·g^−1^, although it was still more than the highest adsorption capacity of many previously published adsorbents when HSA was not present. MIL-100 (Fe) also offers good reusability and facile desorption in addition to its high adsorption capacity. About 1 mL of methanol can desorb 80% of the adsorbed creatinine from MIL-100 (Fe) under sonication for 5 min (Figure 10D(a)). Repeating this regeneration step three times resulted in the desorption of 97.6% of creatinine (Figure 10D(b)). The renewed MIL-100 (Fe) may be repeatedly employed for creatinine adsorption without significant loss of adsorption capacity, and the MIL-100 (Fe) skeleton did not collapse during repeated adsorption of creatinine and regeneration of methanol. In conclusion, MIL-100 (Fe) has a great perspective for the adsorption and removal of uremic toxins and deserves further examination.

#### 3.1.3. Other Types of MOFs

Bio-MOFs are composed of metal ions and biomolecular ligands (sugars, peptides, amino acids, nucleobases, and proteins) [109]. As a sustainable framework for development, bio-MOFs have shown many applications in the biomedical field and have attracted extensive attention [110,111,112,113,114]. Previous studies have shown that MOF membranes containing amino acids and zinc on uremic toxin are highly selective [115]. In order to accurately screen the optimal MOF structure that can efficiently remove PBUTs, the researchers used the Giant Classical Monte Carlo (GCMC) simulation and calculation method [116,117,118]. Yıldız et al. evaluated the adsorption behavior of 315 bio-MOF for urea, creatinine, water, and their mixtures by this method [107]. The results showed that adenine-based bio-MOF, including Bio-MOF-11 (YUVSUE) and Bio-MOF-12 (BEYSEF), and dicyandiamide-based MOF (KEXDIB) are potential adsorbents for the removal of urea/water and creatinine/water mixtures, respectively. In addition, bio-MOF can be used as an alternative to dialysis membranes in dialysis devices with the potential to separate uremic toxins. Palabıyık et al. combined GCMC and equilibrium molecular dynamics (EMD) simulations to predict the separation performance of 60 bio-MOF membranes for urea/water and creatinine/water mixtures [115]. The results showed that carboxylate-based MOF (OREZES) and amine-based MOF (BEPPIX) were the most selective membrane materials for the separation of urea/water and creatinine/water, respectively. The structures of five common bio-MOFs, BEPNIV, BEPPIX, KEXDIB, OREZES, and YUVSUE, were identified as promising candidates for urea/water and creatinine/water infinite dilution separations [115]. In addition, Li et al. conducted a comprehensive screening of bio-MOFs with high efficiency in adsorbing IS and utilized the GCMC model to calculate the adsorption capacity of the selected MOFs for IS [119]. The results showed that aromatically coordinated MOFs with both carboxylic acid groups and metal clusters performed best on IS adsorption (>2100 mg/g). This high adsorption is attributed to the combined effect of the negatively charged carboxylic acid group, the lone pair of electron-containing pyrrolidine nitrogen atoms, and the open metal active site.

Uremic toxins exist as a kind of salt. Cationic MOFs with exchangeable anions exhibit excellent adsorption performance in eliminating inorganic anions [120,121,122,123,124]. Based on this, Zhang et al. constructed cationic MOFs of ZJU-X6 and ZJU-X7 (Zhejiang University, Xiao’s group) by using tetra (4-ethylphenyl) ethylene as the ligand skeleton, the pyridyl unit as the functional group, and nickel/silver nitrate as the metal node [125]. These two cationic MOFs have a high adsorption capacity and good adsorption kinetics. They can efficiently capture PBUTs by anion exchange with the help of hydrogen bonding and hydrophobic contact between the guest toxin molecules and MOF materials. The adsorption capacities of ZJU-X6 for pCS and IS were about 197.2 mg·g^−1^ and 230.4 mg·g^−1^, respectively. The capacity for the adsorption of ZJU-X7 for pCS and IS were 57.0 mg·g^−1^ and 118.6 mg·g^−1^, respectively. The adsorption ability and adsorption kinetics of ZJU-X6 were faster than most of the reported materials.

**Figure 11:**
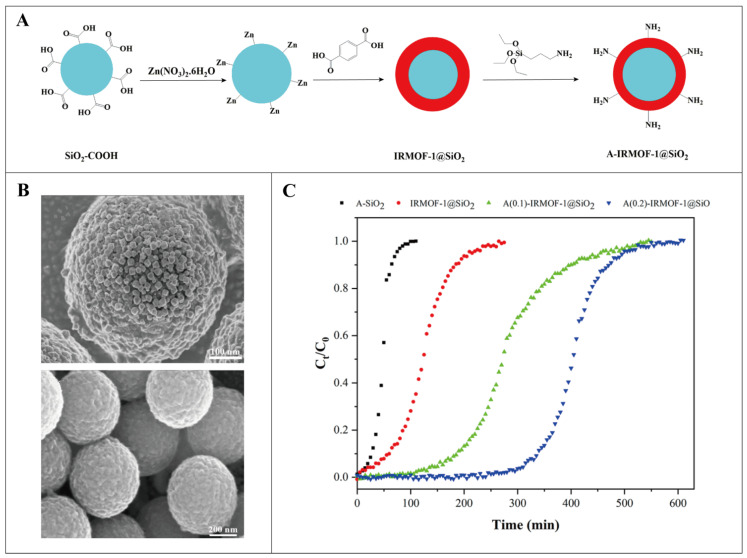
(**A**) Schematic diagram of the synthesis of IRMOF-1@SiO_2_ core-shell of amine functionalization. (Blue represents the core, red represents the shell.) (**B**) IRMOF-1@SiO_2_ SEM diagram, element mapping diagram. (**C**) IRMOF-1@SiO_2_ adsorption isotherm for creatinine. Reproduced from ref. [129] with permission from American Chemical Society, copyright 2023.

Isoreticular MOFs (IRMOFs) or IRMOF-1, also denoted as MOF-5, is a metal-organic network composed of Zn_4_O clusters with different carboxylate linkers, which has a strong ability to adsorb organic materials [126,127]. IRMOF-1 is known for its outstanding strength, flexibility, extremely porous and ordered structure, good thermal solidity, and flexibility of functional moieties [128]. To remove some small toxin molecules, including creatinine and urea, which are difficult to remove during hemodialysis, Hossein et al. developed an efficient adsorbent, amino-functionalized A(0.2)-IRMOF-1@SiO_2_ fixed-bed chromatographic column [129], as shown in Figure 11. The adsorption capacity of the adsorbent was seen to be 1325.73 mg·g^−1^ for urea and 625.00 mg·g^−1^ for creatinine, indicating a strong adsorption impact on these two substances. The urea and creatinine separation coefficients were 2.40%, 92.57%, and 80.47%, respectively, eliminated using A(0.2)-IRMOF-1@SiO_2_ fixed bed column. When choosing an adsorbent with the highest adsorption capacity, one of the most crucial factors is whether or not it has amino groups on its surface.

**Figure 12:**
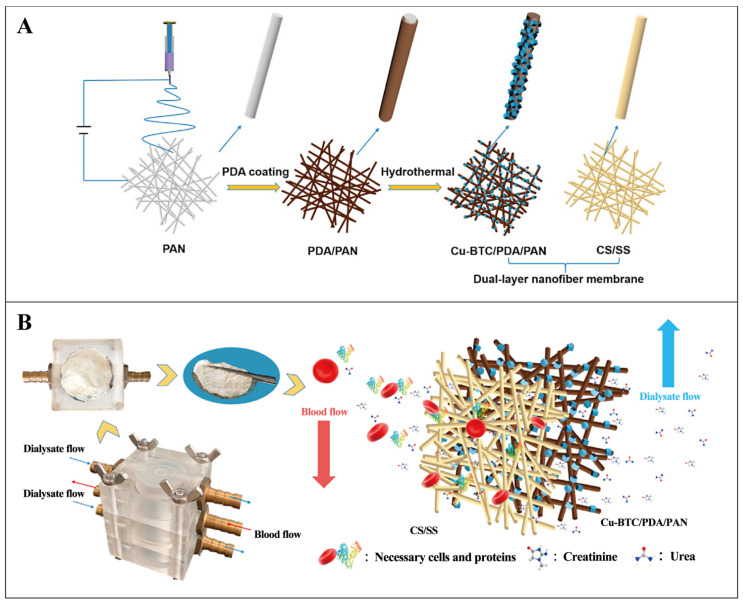
(**A**) Schematic diagram of the preparation process of the double-layer composite nanofiber film. (**B**) Pictorial representation of the process of dialysis. Reproduced from ref. [131] with permission from Elsevier, Copyright 2022.

It has been reported that the adsorption capacity of urea on dry Cu-based MOF (Cu_3_(BTC)_2_, BTC: 1,3,5-phenyltricarboxylate) may reach 250 mg·g^−1^ [130]. Li et al. prepared a unique nanofiber membrane hybrid with a double-layer assembly for use as a hemodialysis membrane by merging electrospun nanofibers with self-assembled MOF components, as shown in Figure 12 [131]. The surface of the first layer contains polydopamine/polyacrylonitrile (PDA/PAN) composite nanofibers specially prepared by Cu-BTC self-assembly modification. The modification of Cu-BTC increases the specific surface area of the nanofiber membrane by nearly two times. The second layer is a chitosan/sericin composite nanofiber biopolymer. The double-layer composite nanofiber membrane effectively adsorbs urea and creatinine. In simulated dialysis experiments, the maximum adsorption was up to 152.44 mg·g^−1^ for urea and 100.50 mg·g^−1^ for creatinine. It is encouraging to note that this dual-layered composite nanofiber membrane not only demonstrated high efficiency in toxin removal but was also equally good at keeping blood components stable. It has good protein retention (83.9% for BSA) and excellent blood compatibility. This means that important proteins in the blood are retained while uremic toxins (e.g., creatinine and urea) are effectively removed. In conclusion, this hemodialysis membrane material has a promising application in artificial kidneys.

Numerous studies have demonstrated the effectiveness of MOFs as uremic toxin adsorbents in dialysis treatment. Table 2 summarizes various MOFs as uremic toxin adsorbents for dialysis treatment of renal diseases, including MOF-based agents, types of adsorbed uremic toxins, adsorption capacity, and their clearance efficiency.

### 3.2. MOFs as a Drug Carrier for the Treatment of Kidney Disease

Compared with other porous-structured nanomaterials, MOFs possess the following many merits [110]: (a) High specific surface area and porosity, which can be used for high-loading therapeutic drugs. (b) It is easy to modify the physical and chemical characteristics of MOFs by the existence of organic ligands or inorganic clusters. (c) Through the open window and pore of MOFs, the diffusion matrix can interact with the binding molecules. (d) Finally, a clear structure is conducive to the study of host-guest interaction. Due to these unique properties, MOFs are excellent carriers for drug delivery.

**Figure 13:**
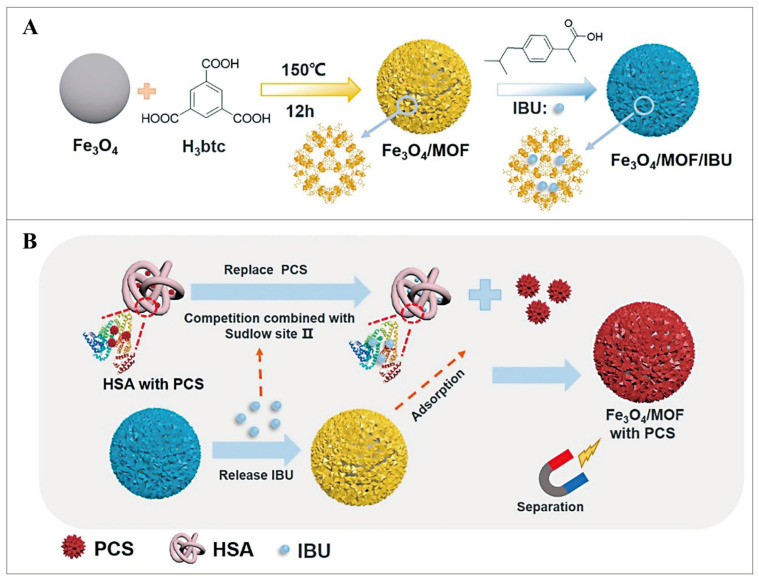
(**A**) Scheme for the preparation method of Fe_3_O_4_/MOF/IBU nanoremover. (**B**) The mechanism of removing PCS in blood. Reproduced from ref. [98] with permission from American Chemical Society, copyright 2022.

Studies have found that some PBUTs can be desorbed reversibly from HSA-PBUTs complexes. They represent that free toxins can be competitively adsorbed by adsorbents [132]. The non-steroidal analgesic ibuprofen (IBU) has the same serum albumin binding location as pCS, and the binding site affinity is greater between IBU and HSA. IBU can effectively replace PBUTs [133,134]. Chen et al. used an in-situ one-step method to encapsulate magnetic Fe_3_O_4_ nanoparticles on the porous MOF MIL-100(Fe) shell [98]. Then, the IBU was filled up into the pores to aquire a Fe_3_O_4_/MOF/IBU nano-removal agent with a core-shell structure with rich pores and good biocompatibility (Figure 13A). The IBU in the Fe_3_O_4_/MOF/IBU nano-remover is released after entering the blood flow and can be competitively adsorbed. Afterward, MOFs bind to free pCS via hydrophobic and classical interactions. Finally, the nano-remover containing pCS was magnetically separated from the blood to achieve efficient removal of PBUTs (Figure 13B). The removal rate of the Fe_3_O_4_/MOF/IBU nano-removal agent was around 26.7% when the initial concentration of pCS in the blood was 100 ppm. PBUTs in the blood can be eliminated using the displacement technology-based magnetic nano-remover, which also offers a dependable method of doing so.

**Figure 14:**
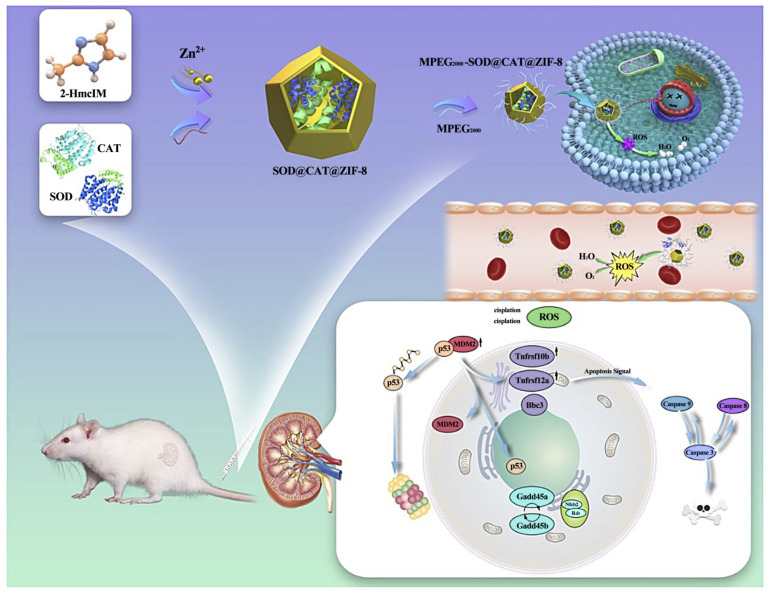
MPEG_2000_SOD@CAT@ZIF-8 (PSCZ) platform clear ROS schematic diagram. Reproduced from ref. [138] with permission from Authors, copyright 2022.

Natural antioxidant enzymes such as superoxide dismutase (SOD) and catalase (CAT) can be used as active oxygen scavengers [135,136], which may reduce excessive oxidative stress. The excessive oxidative stress is caused by excessive reactive oxygen species (ROS) in the serum of patients with acute kidney injury (AKI) [137]. It plays a therapeutic role in AKI. Hou et al. used the biomimetic mineralization approach to compress CAT and SOD in ZIF-8 and then fixed with MPEG_2000_-COOH to obtain a more stable and biocompatible MPEG_2000_-SOD@CAT@ZIF-8 (PSCZ) composite [138]. The transport of intracellular enzymes and the antioxidant effect were greatly enhanced when the composite material was employed as a stabilizing agent with antioxidant qualities for the cascade-based AKI comprehensive treatment. This MOF with dual-enzyme embedding allows for the co-delivery of SOD and CAT enzymes, which are efficient scavengers of reactive oxygen species (Figure 14). When it came to protecting mice from akik-related oxidative renal tissue damage, the integrated MPEG_2000_SOD@CAT@ZIF-8 (PSCZ) platform outperformed free CAT and SOD in terms of SOD and CAT enzymatic efficiency in vitro and improved ROS scavenging capabilities in vivo. To summarize, ‘plating’ based on ZIF-8 is an efficient enzyme protection technique that has a higher therapeutic efficacy and can help determine the exact medical treatment for AKI.

**Figure 15:**
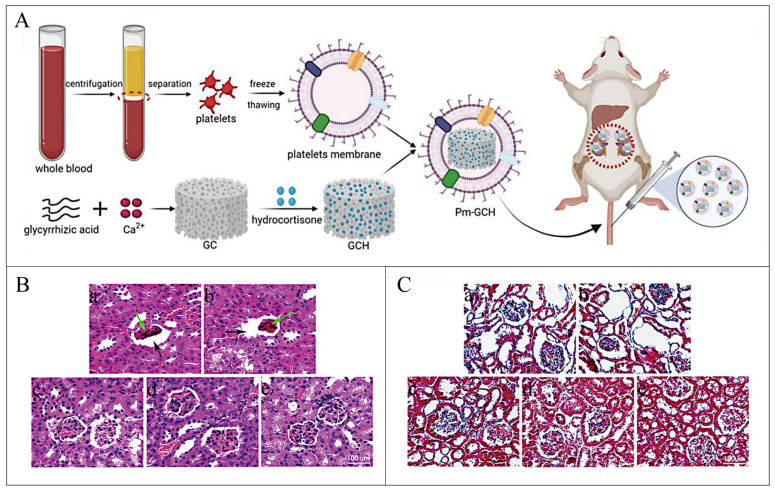
(**A**) Schematic diagram of Pm-GCH synthesis. Renal representative images with H&E staining (**B**) and Masson staining (**C**) after intravenous administration of PBS(a), GC(b), H(c), GCH(d), and Pm-GCH(e), respectively (black arrows, expanding the space of Bauman; green arrow indicates glomerular sclerosis). Reproduced from ref. [139] with permission from Authors, copyright 2021.

Li et al. developed a new core-shell nanoparticle drug system (Pm-GCH) for the action of steroid-resistant nephrotic syndrome (SRNS) by using the MOF (GC). This MOF was prepared by glycyrrhizic acid (G) and Ca^2+^-filled hydrocortisone (H) as the core of nanostructures and platelet membrane vesicles as the shell [139], as shown in Figure 15. Pm-GCH can target renal inflammatory areas non-specifically because of its design, which also confers superior biocompatibility and immune escape capabilities. Hydrocortisone and glycyrrhizic acid are released when GCH gradually breaks down in an inflammatory milieu. When used to treat steroid-resistant nephrotic syndrome (SRNS), glycyrrhizic acid inhibits the inactivation of hydrocortisone, blocks the creation of inflammatory factors, inhibits the activity of phospholipase A2 (PLA2), and complements C2 classical activation trail, and increases the effectiveness of hydrocortisone. Treating SRNS with Pm-GCH is a potentially effective approach.

### 3.3. Other Treatment Methods

Studies have shown that the metabolism of trace elements (Zn, Se, and Fe) in patients with CKD is unbalanced [140,141,142,143,144,145]. By chelating or supplementing appropriate changes in the metabolism of these elements, it can improve the condition and beneficial for CKD [146,147,148,149]. Nano-chelation technology provides a unique way to synthesize nanostructures containing trace elements, which has the potential to improve the multiple dysfunctions of chronic diseases [150,151,152,153].

A chromium-containing MOF (DIFc) prepared using nano-chelation technology demonstrated satisfactory efficacy in evaluating its impact on the biochemical indicators of diabetic rats and parameters related to CKD [154]. In urine samples, DIFc treatment can decrease albumin, malondialdehyde, and 8-iso-prostaglandin while raising the creatinine clearance rate. In plasma samples, DIFc treatment can decrease the HOMA-IR index, blood urea nitrogen, uric acid, and malondialdehyde. Related studies have shown that chromium supplements such as DIFc may have a protective effect on the kidney through antioxidant and anti-inflammatory effects [149,155,156]. In addition, Fakharzadeh et al. synthesized another MOF (DIBc) containing selenium, zinc, and chromium using nano-chelation technology [157]. DIBc has iron-chelating properties, and its anti-diabetic effect has been evaluated [158]. Through a study involving rats administered a high-fat diet and induced with streptozotocin to mimic Diabetic nephropathy (DN), the research team meticulously evaluated the efficacy of DIBc in ameliorating crucial biochemical and structural markers associated with CKD [157]. The findings highlighted the potential of DIBc in enhancing a range of CKD indicators. The CKD indicators encompassed blood glucose regulation, reduction in urea nitrogen and uric acid concentrations, decrease in malondialdehyde levels indicative of oxidative stress, enhancement in the Homeostatic Model Assessment for Insulin Resistance (HOMA-IR) index, attenuation of the urinary albumin excretion rate, and mitigation of glomerular basement membrane alterations. These results collectively underscore the promise of DIBc as a therapeutic intervention for addressing multifaceted aspects of CKD pathology.

## 4. Conclusions, Challenges, and Prospects

The continuous development of MOF-related research delivers new options for the diagnosis and treatment of kidney diseases, showing significant advantages and potential. However, its application still faces a series of problems and challenges: (a) The detection of biomarkers requires sensors with high specificity and sensitivity. Urine, blood, tears, and other biological samples contain a variety of components, which may interfere with the accurate detection of the sensor. Therefore, it is necessary to develop more advanced sensor design and surface modification strategies to improve anti-interference ability. (b) MOF-based sensors typically detect only a single biomarker. Therefore, it is a promising research direction to integrate the characteristics of various biomarkers and develop multifunctional MOF sensors capable of simultaneously detecting multiple biomarkers, thus enhancing the accuracy and comprehensiveness of diagnosis. (c) Currently, research on MOFs and their compounds for the diagnosis and treatment of kidney disease is still in the laboratory stage. It should be validated through large-scale clinical trials and practical application to evaluate potential biological toxicity, immune response, and long-term exposure in vivo, providing stronger support for widespread application. (d) The adsorption process is a dynamic equilibrium process, which requires the dialysis membrane with high stability and durability. Furthermore, prolonged use may lead to membrane contamination by substances such as blood and proteins. Therefore, in practical applications, ensuring the stability and durability of its performance and effectively cleaning and preventing pollution are problems that need to be solved. (e) While MOFs have shown potential in hemodialysis, further investigation is required to evaluate the impact of different types of MOFs on dialysis efficiency and to develop optimal strategies for their application in this context.

Although the emerging MOF-based diagnosis and treatment of kidney diseases face a series of problems and challenges, there are still many opportunities for its application to grow. With the continuous growth and innovation of science and technology, the current limitations and challenges of developing more efficient, safe, and multifunctional MOF nano-diagnostic materials are expected to be overcome in the future. In general, the diagnosis and treatment of kidney diseases based on MOFs is an area full of opportunities and challenges. In the future, with the continuous development and improvement of related technologies, this field is expected to make more breakthroughs and progress.

## Figures and Tables

**Table 1:** Advantages and limitations of various agents for MOFs-based diagnosis of kidney disease.

Biological Sample	MOF-Based Agents	Biomarkers Detected	Advantages	Limitations	Ref.
Urine	Au@MIL-101(Fe)	Creatinine	Non-invasive. Low detection limit. Wide linear response range.	Lack of long-term stability studies. Lack of reusability studies. Lack of adequate clinical validation.	[35]
	Mag Zr-MOF@G6P	Glyco- proteins	Non-invasive. Low detection limit. Good selectivity.	Complex recovery process. Limited enrichment capacity.	[43]
	Fe_3_O_4_@NH_2_-MIL- Ti@TTA-MA	Glyco- and phospho- proteins	Non-invasive. Low detection limit. Good selectivity. Good reusability. Synergistic.	Inadequate sample representation. Lack of long-term stability studies. Lacks adequate clinical validation.	[44]
	Ti-MOF-MO	Specific metabolites (Arg, His, Glu)	Non-invasive. Low detection limit. Low sample consumption. High diagnostic accuracy.	Not portable. High cost of synthesis. Lack of metabolite database support. Lack of adequate clinical validation.	[49]
Breathe gas	ZENose (Fc@ZIF-8)	Ammonia	Non-invasive. Low detection limit. Wide linear response range. Remote Point of Care (POC).	Electrode regeneration is unknown. Lack of long-term stability studies. Lack of adequate clinical validation.	[62]
Blood	UiO-HQCA-Al	Creatinine	Low detection limit. Wide linear response range. Fast response. High selectivity.	Environmentally sensitive. Lack of adequate clinical validation.	[67]
Tears	Cu-BDC MOF/GO- Cu(II)/Cu_2_O NPs	Creatinine	Non-invasive. High selectivity and sensitivity. Precise predictive capability. Remote Point of Care (POC).	High cost of synthesis. Lack of adequate clinical validation. Deficiencies in the generalization ability of machine learning models.	[71]

**Table 2:** Application of MOFs as uremic toxin adsorbent in the treatment of kidney disease.

MOFs Classification	MOFs and Their Composites	Uremic Toxins	Adsorption Capacity (mg·g^−1^ MOF)	Removal Efficiency (%)	Ref.
Zr-based MOFs	NU-1000	pCS	294.9	94	[81]
		IS	Not available	98	[81]
	UiO-66	HA	Not available	2.1	[81]
	PPNUH	pCS	282.0	85	[88]
		IS	329.0	85	[88]
	UiO-66-NH_3_ (75%)	IS	Not available	80	[90]
		HA	Not available	83	[90]
	UiO-66-NH_2_ (75%) 12.5% HCl	IS	Not available	80	[90]
		HA	Not available	77	[90]
	UiO-66-(COOH)_2_@ cotton fabric	Creatinine	212.8	98	[95]
	UiO-66-(COOH)_2_@PAN (UAPNFM)	Creatinine	168.6	82	[97]
	UiO-66-(COOH)_2_@ PVA/PAN TFNC	Creatinine	54.0	Not available	[98]
Fe-based MOFs	MIL-53(Fe)	Urea	635.0	96	[103]
	MIL-100(Fe)	Urea	692.0	97	[103]
		pCS	12.9	65	[106]
		Creatinine	190.5	89	[108]
Bio-MOFs	Bio-MOF-11 (YUVSUE)	Urea	38.7	Not available	[107]
	Bio-MOF-12 (BEYSEF)	Urea	63.6	Not available	[107]
	Methionine-derived MOF (OREZES)	IS	2100.0	98	[115]
		Urea	347.9	Not available	[115]
Cationic MOFs	ZJU-X6	pCS	197.2	98	[125]
		IS	230.4	94	[125]
	ZJU-X7	pCS	57.0	78	[125]
		IS	118.6	97	[125]
Isoreticular MOFs (IRMOFs)	A(0.2)-IRMOF-1@SiO_2_	Urea	1325.7	92	[129]
		Creatinine	625.0	80	[129]
Cu-based MOFs	Cu_3_(BTC)_2_	Urea	250.0	Not available	[130]
	Cu-BTC@PDA/PAN nanofiber	Urea	152.4	92	[131]
		Creatinine	100.50	82	[131]

## Data Availability

Not applicable.
